# The Use of Segmental and Suprasegmental Sequencing Skills to Differentiate Children With and Without Childhood Apraxia of Speech: Protocol for a Comparative Accuracy Study

**DOI:** 10.2196/40465

**Published:** 2022-10-04

**Authors:** Min Ney Wong, Eddy C H Wong, Shelley L Velleman

**Affiliations:** 1 Department of Chinese and Bilingual Studies The Hong Kong Polytechnic University Hong Kong SAR Hong Kong; 2 Research Centre for Language, Cognition, and Neuroscience The Hong Kong Polytechnic University Hong Kong SAR Hong Kong; 3 Research Institute for Smart Ageing The Hong Kong Polytechnic University Hong Kong SAR Hong Kong; 4 Department of Communication Sciences and Disorders The University of Vermont Burlington, VT United States

**Keywords:** childhood apraxia of speech, diagnosis, pitch variation

## Abstract

**Background:**

Childhood apraxia of speech (CAS) is a motor-based speech sound disorder (SSD) with a core impairment in the planning and programming of spatiotemporal parameters of speech movement sequences. CAS may cause deficits in both segmental and suprasegmental components of speech, and it can severely affect children’s ability to speak intelligibly and communicate effectively and impact their quality of life. Assessment tasks, such as the maximum performance tasks (MPT) and Syllable Repetition Task (SRT), examine children’s segmental sequencing skills to assist with the diagnosis of CAS. In Hong Kong, although the MPT and SRT have been used clinically to diagnose CAS in Cantonese-speaking children, their validity has not been reported. There is an urgent need for such investigations. Suprasegmentally, lexical stress errors have been reported as a consensual feature and to aid in the diagnosis of CAS. However, there are challenges in diagnosing CAS in children who speak tonal languages like Cantonese. A recent study has reported lexical tone errors in Cantonese-speaking children with CAS. Furthermore, deficits in pitch-variation skills were found in Cantonese-speaking children with CAS using a tone sequencing task (TST). It is hypothesized that there is a universal deficit in pitch-variation skills among tonal and nontonal language speakers with CAS. Further investigations of pitch-variation skills using the TST in Cantonese-speaking children with CAS may shed light on suprasegmental deficits in tonal languages and contribute to the development of a valid diagnostic tool for CAS in children who speak other tonal languages, such as Vietnamese, Thai, and Mandarin.

**Objective:**

This study aims to examine the diagnostic potential of the MPT, SRT, and TST in diagnosing Cantonese-speaking children with CAS and to investigate pitch-variation skills in Cantonese-speaking children with and without CAS.

**Methods:**

A total of 25 children with CAS and 3 groups of age- and gender-matched controls (non-CAS SSD only group, non-CAS SSD co-occurring with language impairment group, and typical development group) will be recruited. All participants will perform the MPT, SRT, and TST measures. Their performances on these tools will be perceptually judged and acoustically measured.

**Results:**

Data collection will last from January 1, 2022, to October 30, 2023. As of August 2022, the project has recruited 4 children in the CAS group, 21 children in the non-CAS SSD group, 4 children in the speech and language impairment group, and 53 children in the typical development group.

**Conclusions:**

It is anticipated that Cantonese-speaking children with CAS will have poorer pitch-variation skills than the control groups and that the MPT, SRT, and TST will be appropriate diagnostic tools for identifying CAS in Cantonese-speaking children. The project will benefit the field of speech-language pathology locally and internationally, with short- and long-term impacts.

**International Registered Report Identifier (IRRID):**

DERR1-10.2196/40465

## Introduction

### Background

Childhood apraxia of speech (CAS) is a motor-based speech sound disorder (SSD) with a core impairment in planning and programming of spatiotemporal parameters of speech movement sequences. It can occur as an idiopathic neurogenic SSD or as a result of known neurological impairment [1]. It can severely affect children’s ability to speak intelligibly and communicate effectively. CAS onsets from childhood and may persist into adolescence and adulthood [2-4]. If left untreated, the quality of life of children with CAS will be affected because of the long-term consequences of CAS on articulation, speech intelligibility, expressive language [5], academic performance, and social functioning [2-4,6,7].

CAS in children is characterized by deficits in both segmental and suprasegmental components of speech. The American Speech-Language-Hearing Association [1] reported that there are three consensual features, including (1) inconsistent errors in sequential repetitions; (2) deficits in coarticulation or syllable segregation (ie, choppy speech); and (3) prosodic deficits, especially lexical stress errors. Lexical stress errors, as one of the consensual features of CAS, have been widely studied in English speakers because of their high value as a suprasegmental marker of CAS [8]. The errors reflect underlying deficits in speech motor planning and programming skills that control the intensity, frequency, and duration of suprasegmental parameters. Deficits in the temporal control of lexical stress production have been documented, but it has been difficult to separate other specific acoustic aspects of lexical stress, such as pitch [9].

### CAS Diagnosis in English-Speaking Children

The gold standard for making a CAS diagnosis primarily relies on perceptual judgments of CAS experts. However, very recently, a pilot study reported only moderate agreement among 4 expert listeners [10]. Along with perceptual judgment, the maximum performance tasks (MPT) [11] and Syllable Repetition Task (SRT) [12] have been reported to provide valuable relevant information about segmental sequencing skills in children with CAS. Both MPT and SRT assess deficits in speech processing. The MPT aims to assess motor involvement in children with speech problems [11]. Specifically, the maximum repetition rate for trisyllabic stimuli (MRRtri) and maximum fricative duration (MFD) in MPT assess underlying deficits in the motor planning and programming of speech [13], while the maximum repetition rate for monosyllabic stimuli and maximum phonation duration assess deficits in speech motor execution. The SRT targets encoding, memory, and transcoding processes, which refer to mapping, prearticulatory or phonological planning, and transformation of the phonological plan into a motor plan, respectively [14]. Although the terminology is not identical, both the MPT and SRT address the same underlying deficits of CAS, speech motor planning and programming skills. Research has shown that the MPT has high sensitivity and specificity (ranging from 89% to 100%) in making a CAS diagnosis [11], whereas a cutoff transcoding score of 80 on the SRT is able to differentiate children with CAS from children with concomitant speech delay and language impairment (LI) [15].

### CAS Diagnosis in Cantonese-Speaking Children

There are challenges in diagnosing CAS in children who speak tonal languages like Cantonese. In Hong Kong, speech-language pathologists may apply English-based research findings to assess, diagnose, and treat children with CAS among the local Cantonese-speaking population. Although MPT and SRT have been used clinically to diagnose CAS in Cantonese-speaking children, the validity of applying these objective measures and diagnostic criteria from English-speaking populations to Cantonese-speaking populations is unknown. Owing to the segmental and suprasegmental differences between English and Cantonese, such as the fact that English has lexical stress patterns while Cantonese has lexical tones, English-based findings cannot be fully applied to Cantonese speakers with CAS, especially as prosodic deficits are one of the consensual clinical features of CAS. The current gold standard relies on expert perceptual judgment of clinical features reported in the English literature. Therefore, it is possible that misdiagnoses or underdiagnoses are occurring in the current clinical practice in Hong Kong. As higher frequency treatment is suggested for children with CAS [16] owing to the need for speech motor learning, inaccurate diagnoses may impact children, families, and society in terms of allocation of resources. Therefore, a valid diagnostic tool is urgently needed for Cantonese speakers with CAS to correctly identify and provide appropriate treatment to children with CAS.

### Previous Pilot Studies

A recent study [14] identified a new clinical feature, namely tone production errors, which has not been reported in English speakers with CAS. The authors proposed that the same underlying deficit of speech motor planning and programming skills manifests differently in Cantonese and English [14]. Both English and Cantonese speakers with CAS may have similar control challenges with the correct production of segments; however, they control suprasegmentals differently because of linguistic differences. Suprasegmentals are overlaid differently for lexical stress in English versus lexical tone in Cantonese. English speakers apply 3 stress features—simultaneous pitch, loudness, and duration variations—to syllables to express a grammatical or pragmatic meaning. Cantonese speakers vary the pitch of segments to indicate different lexical meanings. Owing to these differences, further investigations of lexical tone errors are vital to increase the understanding of tone production skills in children with CAS.

In an effort to further explore tone production skills in Cantonese-speaking children with CAS, tone sequencing tasks (TSTs) have been used in 2 studies to examine the accuracy, consistency, and duration of tone production in Cantonese-speaking children with CAS [17,18]. The TST is considered to be a potential assessment task that reflects impairment in speech motor planning and programming in Cantonese-speaking children with CAS [17,18]. It requires children to produce 5 repetitions of each item that is formed of 3 early-acquired Cantonese tones, that is, tone 1 (high-level), tone 2 (high-rising), and tone 4 (low-falling). Wong et al [18] administered the initial version of the TST to 2 Cantonese-speaking children with CAS and 2 Cantonese-speaking children with non-CAS SSDs. The results showed that children with CAS performed significantly more poorly than children without CAS in sequencing tones. The effect sizes (Cohen *d*) for tone accuracy and consistency were 0.68 and 0.60, respectively. Both of them were considered to be medium-to-large effect sizes.

In a subsequent study, the research team examined the linguistic effects of syllable structure and lexical status on TSTs using the second version of the TST [18]. A total of 4 Cantonese-speaking children with CAS were matched with 3 children with non-CAS speech and LI (S&LI), 3 with LI alone, and 3 with typical development (TD). Tone accuracy was judged perceptually and calculated as the percentage of tones correct. The consistency strength procedure adopted from the study by Williams et al [19] was used to calculate consistency scores. In the study by Wong et al [20], the team added 2 acoustic measures (ie, fundamental frequency [F0] values and acoustic duration) to further examine the data. The results showed both syllable structure and lexical status effects. Cantonese-speaking children with and without CAS showed significant between-group differences in F0 values for both vowel and consonant-vowel structures, as well as tone accuracy, tone consistency, and acoustic duration of word stimuli. A small to medium effect size (Cohen *d* ranging from 0.22 to 0.61) was obtained for F0 values, whereas large effect sizes were obtained for tone accuracy (Cohen *d* ranging from 1.37 to 1.98), tone consistency (Cohen *d* ranging from 1.36 to 1.85), and acoustic duration (Cohen *d* ranging from 0.9 to 1.45). The results suggest that syllable structure and lexical status play some roles in tone sequencing skills in children with CAS. In summary, these pilot studies suggest that children with CAS have difficulty with pitch-variation skills, specifically tone sequencing skills, and perform more poorly than control groups. The findings also support the diagnostic potential of TST as it provides a platform to investigate pitch variation in children with CAS and should be further developed as a linguistically appropriate assessment tool for Cantonese-speaking children. However, the generalizability of these findings is limited because of the extremely small sample sizes.

### Studying Pitch-Variation Skills Using the TST

Pitch-variation skills refer to the skills of varying F0 values within and between syllables in tonal languages. Pitch-variation skills present in English lexical stress patterns and Cantonese lexical tones. Although lexical stress errors have been empirically studied and it has been found that children with CAS have a deficit in the temporal control of lexical stress productions [9], the development and disorders of pitch-variation skills embedded in lexical stress errors remain unknown. Kopera and Grigos [9] attempted to examine the acoustic properties (ie, duration, peak F0, and average F0) of lexical stress errors using the pairwise variability index [21] between children with and without CAS. No significant between-group difference was found. The authors concluded that lexical stress errors in children with CAS can only be studied in a collective fashion, such as using the lexical stress ratio [8], which examines pitch, loudness, and duration simultaneously, instead of investigating acoustic parameters independently.

Nevertheless, new findings have shown significant between-group differences in both F0 values and acoustic duration between children with CAS and control groups [20]. This preliminary result suggests that Cantonese-speaking children with CAS have difficulty in varying pitch and that pitch-variation skills in Cantonese speakers with CAS can be studied independently via the TST in the context of this tonal language. This provides the basis for further investigations of pitch-variation skills in children with CAS who speak other tonal languages (eg, Mandarin, Vietnamese, and Thai) as well as children with CAS who speak nontonal languages (eg, English). TST can be used in English to assess pitch-variation skills in children with CAS in an out-of-stress context. This application may elucidate the role of pitch control in out-of-stress contexts and shed light on pitch control across languages in speakers with CAS.

### The Goal of the Proposed Study

This study aims the following:

1. To show that the MPT and SRT can contribute to the diagnosis of CAS in Cantonese-speaking children.
2. To document differences in pitch-variation skills in Cantonese-speaking children with versus without CAS.
3. To prove that TSTs are effective in diagnosing CAS in Cantonese-speaking children.

We hypothesized the following:

1. The MPT and SRT would differentiate Cantonese-speaking children with CAS from those without CAS.
2. Cantonese-speaking children with CAS would have significantly poorer pitch-variation skills than the control groups.
3. TSTs would differentiate Cantonese-speaking children with CAS from those without CAS.

## Methods

### Protocol Version

The original version of the protocol was submitted to the Research Grants Council of the Hong Kong Special Administrative Region Government on October 30, 2020. This protocol is version 2, in which the sample size was changed from 120 to 100 children. This change was approved by the Research Grants Council on May 17, 2022.

### Study Design

This is a comparative accuracy study that will compare the diagnostic accuracy of the MPT, SRT, and TST in children with and without CAS.

### Participants

A total of 25 children with CAS will be recruited for the study. The inclusion criteria are as follows: (1) age between 3 years and 6 years 11 months, (2) diagnosed with or suspected of having CAS by a qualified speech-language pathologist, (3) no hearing impairment or structural abnormality that affects speech production, and (4) Cantonese as the main language for daily communication. The same number of age- and gender-matched children will be recruited for each of the following 3 control groups: non-CAS SSDs only, non-CAS SSDs co-occurring with S&LI, and TD. The non-CAS SSD group will be recruited because it will allow a direct comparison between children with and without CAS. Research has shown that LI is usually present in children with CAS [5]; therefore, an S&LI group will be included to compare the performance of children with and without CAS, while controlling for language skills. A total of 100 participants will be recruited. Originally, a sample size of 172 participants (ie, 43 for each group) was estimated from the effect size of tone accuracy in a pilot study [20] via power analysis. An actual power of 0.954 could be achieved from 172 participants with an error probability of 0.05 (*F*_168_=2.658). There should be sufficient children with CAS in this population. However, taking into consideration the time and effort needed to screen and find suitable participants with and without CAS, SSD, and S&LI, it will be very challenging to recruit 172 participants within 2 years (the proposed study period). In addition, many reported studies [22,23] have only recruited 20 to 30 children with CAS for the investigations of assessment and diagnostic accuracy. Therefore, the total sample size was reduced to 100 participants.

### Recruitment

A recent study estimated the population-based prevalence of CAS in children aged 4 to 8 years to be 1 child per 1000 [24]. Given that there were about 303,000 children between the ages of 5 and 9 years in Hong Kong in the mid-2020 [25], there may be 303 children in this age range in Hong Kong who are currently impacted by this severe motor speech disorder. Children were recruited through local advertising and invitations. Recruitment posters were posted in the university campus and on the web via social media platforms. The digital version of the posters was posted on the official Facebook page of the university speech-language therapy clinic, the Facebook page and Instagram accounts of Cantonese CAS, and the personal Facebook and Instagram accounts of the members of the research team. For the CAS group, recruitment information was also delivered to speech-language pathologists who attended local continuing education seminars on Cantonese CAS. Personal invitations were sent to the parents of children with CAS who have connections with the research team. The non-CAS groups were recruited from the general public by forwarding the digital posters to the parents of preschool children on WhatsApp chat groups, inviting parents of the children who were receiving speech-language therapy services in the university clinic, and sending invitation emails to kindergartens located in the same district as the university.

### Procedure

#### Initial Assessment

An expert speech-language pathologist will conduct initial assessments to diagnose each participant, if appropriate. The expert speech-language pathologist will have at least 10 years of clinical experience in assessing and treating Cantonese-speaking children with and without CAS. The assessment tasks include case history, speech sample collection, standardized language tests (ie, Hong Kong Test of Preschool Oral Language [Cantonese] [26] and Hong Kong Cantonese Receptive Vocabulary Test [27]), standardized articulation test (ie, Hong Kong Cantonese Articulation Test [28]), imitation of polysyllabic words, a standardized tone identification test (ie, Cantonese Tone Identification Test [29]), an oral and speech motor control assessment [30], and the documentation of prosodic characteristics. If a child is suspected to have autism spectrum disorder (ASD), the expert speech-language pathologist will conduct further assessments. According to Tierney et al [31], there is high comorbidity between CAS and ASD. The diagnosis of CAS and ASD may be delayed or inaccurate when both conditions are present in a child; children with CAS may be wrongly diagnosed with ASD and vice versa. Therefore, the Autism Diagnostic Observation Schedule, Second Edition [32], a standardized tool with high specificity and sensitivity for diagnosing ASD [33], will be administered to obtain information about the appropriateness of an ASD diagnosis. The assessment session will be audio- and video recorded so that another expert speech-language pathologist can review the assessments and diagnose independently. The CAS diagnosis will be confirmed if both speech-language pathologists reach a consensus on the presence of CAS features.

The diagnosis of CAS will be confirmed based on international standards and the methods used in our previous pilot study [20]. A CAS diagnosis will be based on the presence of 3 consensual features [1] and 4 clinical features that have reported 91% diagnostic accuracy [22], with appropriate modifications for Cantonese-speaking children, and across different assessment tasks (eg, speech sample, imitation of polysyllabic words, standardized articulation test, and diadochokinetic tasks). Murray et al [22] suggest using (1) syllable segregation, (2) lexical stress matches, (3) percent phonemes correct from polysyllabic words, and (4) articulatory accuracy on repetitions of [pʌtʌkʌ] for the differential diagnosis of CAS. Modification of some of these features is necessary for Cantonese. The second feature will be changed to *lexical tone errors* owing to the prosodic differences between English and Cantonese. The third feature will be changed from both segmental and suprasegmental correctness to only segmental correctness because of the constant duration of Cantonese syllables [34]. This set of criteria was used in our previous pilot study [20].

#### MPT and SRT

Every child will perform the MPT, SRT, and TST. The order of administration will be randomized. The administrative procedures and interpretation of MPT and SRT are described in the studies by Rvachew et al [35] and Rvachew and Matthews [15], respectively. There are four tasks in the MPT, including (1) maximum phonation duration, (2) MFD, (3) maximum repetition rate for monosyllabic stimuli, and (4) MRRtri. A total of 6 scores can be obtained from these tasks [11]. A dyspraxia score of 0, 1, or 2 is obtained from the performances of MFD and MRRtri. A dyspraxia score of 2 indicates the presence of CAS in children.

The SRT includes 18 items [12]. The items are formed of early developing phones (eg, [m], [d], [n], and [a]), which are easier for younger or severely impaired children to produce. The items included eight 2-syllable stimuli (eg, [bada]), six 3-syllable stimuli (eg, [bamana]), and four 4-syllable stimuli (eg, [bamadana]). The SRT gives 4 scores, including a competency score, an encoding score, a memory score, and a transcoding score. The interpretation of these 4 scores is based on z-scores from the means and SDs reported in the study by Lohmeier and Shriberg [36]. Table 1 presents a comparison of MPT and SRT.

Most of the stimuli used in the MPT and SRT are shared between English and Cantonese. For example, the vowel [a] and initial consonants [m], [f], and [s] in the MPT and SRT are shared in Cantonese. Although the voiced consonants [b] and [d] and voiceless consonants [p], [t], and [k] in English cannot be found in Cantonese, these sounds can be replaced by voiceless unaspirated [p] and [t] and voiceless aspirated [p^h^], [t^h^], and [k^h^] in Cantonese. This replacement is logical as, from a motor perspective, the contrastive aspiration feature in Cantonese consonants is similar to the voicing feature in English consonants [38]. With this logical replacement, it is anticipated that the SRT and MPT will be useful in differentiating between Cantonese speakers with and without CAS, as has been proven in English and Dutch speakers [11,15].

**Table 1:** Comparison of the maximum performance tasks (MPT) and Syllable Repetition Task (SRT).

	MPT [11]	SRT [12]
Area assessed	Motor planning and programming of speech	Encoding process (mapping) Memory process (prearticulatory or phonological planning) Transcoding process (transformation of the phonological plan into a motor plan)
Scores	MPD^a^: mean duration of the longest prolongation of [a] and [mama] MFD^b^: mean duration of the longest prolongation of [s], [f], and [z] MRRmono^c^ score: mean repetition rate for the fastest repetition of each [pa], [ta], and [ka] MRRtri^d^ score: number of syllables produced per second in the child’s fastest repetition of [pʌtʌkʌ] Sequence score: 1 for correct sequence and 0 for unsuccessful production Attempt score: number of attempts required to produce the correct sequence	Competency: PCC^e^ of 18 items Encoding: percentage of consonants within-manner class substitution errors (excluding voicing errors) Memory: ratio of PCC for 3-syllable items to PCC for 2-syllable items Transcoding: percentage of items that are produced with ≥1 additions
Interpretation of the scores	MFD and MRRtri scores are used to obtain a dyspraxia score (0, 1, or 2), while MPD and MRRmono scores are used to obtain a dysarthria score (0, 1, or 2)	Interpretation of these 4 scores is made based on the z-scores from the means and SDs reported in the study by Lohmeier and Shriberg [36]
Criteria of CAS^f^ diagnosis	A dyspraxia score of 2 indicates the presence of CAS; it is obtained when MRRtri ≤3.4 or Sequence=0 or Dyspraxia score is not 0 or 1	Cutoff scores for CAS diagnosis [37]: Competency score=65 Encoding score=46.9 Memory score=67.5 Transcoding score=80

^a^MPD: maximum phonation duration.

^b^MFD: maximum fricative duration.

^c^MRRmono: maximum repetition rate for monosyllabic stimuli.

^d^MRRtri: maximum repetition rate for trisyllabic stimuli.

^e^PCC: percentage of consonants correct.

^f^CAS: childhood apraxia of speech.

#### TST Procedure

The TST proposed in this study is a shorter version modified based on the findings in the study by Wong et al [18]. This new version of TST has 16 stimuli, derived from 2 vowel structures, 2 consonant-vowel structures with early acquired sounds, and 3 early acquired Cantonese tones. The details of the TST are listed in Table 2. There are twelve 1-syllable items and four 3-syllable items. Both word and nonword stimuli are included. The participants will repeat each item as fast as they can 5 times. Four outcome measures will be obtained from the TST:

1. Tone accuracy will be calculated from perceptual judgments of correctness.
2. Tone consistency will be calculated using the consistency strength formula described in the study by Williams et al [19]. The 5 repetitions of each stimulus will be compared with the children’s own baseline one by one to determine the consistency strength of that production.
3. F0 values will be measured using Praat. F0 will be estimated at 5 evenly spaced time points (0%, 25%, 50%, 75%, and 100%) from the beginning to the end of the voiced segment of each syllable in the TST [39].
4. Acoustic durations will be measured in Praat from the onset of the first syllable to the end of the last syllable.

All assessment sessions will be conducted at a local university clinic or laboratory, and all data collection sessions will be conducted in the soundproof booth in the laboratory. Children’s performances on the MPT, SRT, and TST will be audio- and video-recorded. Two speech-language pathologists with experience in childhood disordered speech will perceptually transcribe children’s productions using narrow transcription and score their performance. Furthermore, 20% of the ratings will be rerated by the speech-language pathologists to determine intra- and interrater reliability.

**Table 2:** Stimuli of tone sequencing task (TST) for the proposed study.

TST type	Structure	
	Vowel	Consonant-vowel
**TSTmono^a^**		
	[a1^b^]^c^×5	[pa1]^c^×5
	[a2^d^]^c^×5	[pa2]^c^×5
	[a4^e^]^c^×5	[pa4]^c^×5
	[u1]×5	[hu1]×5
	[u2]×5	[hu2]×5
	[u4]×5	[hu4]×5
**TSTtri^f^**		
	[a1a2a4]×5	[pa1pa2pa4]×5
	[u1u2u4]×5	[hu1hu2hu4]×5

^a^TSTmono: tone sequencing task for monosyllabic stimuli.

^b^The number 1 indicates high-level tone in Cantonese.

^c^Indicates word stimuli (the others are nonword stimuli).

^d^The number 2 indicates high-rising tone in Cantonese.

^e^The number 4 indicates low-falling tone in Cantonese.

^f^TSTtri: tone sequencing task for trisyllabic stimuli.

### Statistical Methods

For the statistical analysis, linear mixed-effects models will be used. Our models will include group (CAS vs SSD vs S&LI vs TD) as a fixed effect and participants and items as random effects.

The sensitivity and specificity of the MPT, SRT, and TST in making a diagnosis of CAS in Cantonese-speaking children will be determined using the receiver operating characteristics curve [40]. The new cutoff scores of the MPT and SRT to diagnose CAS in Cantonese-speaking children will be compared with the existing cutoff scores recommended for English-speaking children. The cutoff scores for the TST will also be determined from the receiver operating characteristics curve.

### Ethics Approval

An information sheet and informed consent form will be given to the parents or guardians of the child participants before the initial assessment or data collection sessions. All parents or guardians will be asked to provide consent by signing the informed consent form given. The parents or guardians of the participants will be informed that their participation is voluntary and that they can withdraw their children at any time without giving a reason and without any negative consequences. All the information provided by the participants and their parents or guardians will be handled confidentially and anonymously, which means that all the data from which the participants can be identified will be removed. All the data will be encrypted and stored in a repository with restricted access. Only researchers working on this study will have access to personal and research data for the purposes of this study. This study has received ethical approval from the Hong Kong Polytechnic University Institutional Review Board (HSEARS 20210125011 and HSEARS 20210330007). Responsible members of Hong Kong Polytechnic University may be given access for monitoring and auditing the research. Any important changes in the protocol will be informed to the Hong Kong Polytechnic University Institutional Review Board.

## Results

Data collection started in January 2022 but was soon disrupted by the fifth wave of the COVID-19 pandemic in Hong Kong. As of August 2022, the project has recruited 4 children in the CAS group, 21 children in the non-CAS SSD group, 4 children in the S&LI group, and 53 children in the TD group. Data collection is ongoing and will continue until October 2023.

## Discussion

### Principal Findings

The proposed study will address an important clinical research gap owing to which there is an urgent need for a valid diagnostic tool for Cantonese speakers with CAS. In particular, we aimed (1) to show that the MPT and SRT can contribute to the diagnosis of CAS in Cantonese-speaking children, (2) to document differences in pitch-variation skills in Cantonese-speaking children with versus without CAS, and (3) to prove that TSTs are effective for diagnosing CAS in Cantonese-speaking children.

### Comparison With Prior Work

With reference to previous investigations of the TST [17,20], it is anticipated that Cantonese-speaking children with CAS will have significantly poorer pitch-variation skills than the control groups. Specifically, Cantonese speakers with CAS will show less variations in F0 values, longer acoustic repetition durations, lower percentages of tones correct, and lower consistency than those in the control groups. It is further anticipated that TST, like MPT and SRT, will be shown to be effective for diagnosing CAS in Cantonese speakers, with appropriate sensitivity and specificity.

### Strengths and Limitations

On completion, this study will provide 2 objective measures (ie, MPT and TST) and 2 measures that convert perceptual judgments into quantitative data (ie, SRT and TST) for diagnosing CAS in Cantonese speakers. The results will promote the standard of CAS diagnosis in Cantonese speakers from reliance on expert perceptual judgment based on a list of clinical features [41] to a combination of perceptual judgment and quantitative data. In addition, the short administration time of the measures proposed in this study (ie, approximately 15-20 minutes per measure) will provide clinicians with quick and accurate methods for CAS diagnosis in Cantonese speakers than approximately 2 hours of comprehensive assessment [18,41,42] of speech motor skills reported in the literature. Moreover, the results of this study will provide the basis for further investigations of pitch-variation skills in children with CAS who speak other tonal languages (eg, Mandarin, Vietnamese, and Thai) as well as in children with CAS who speak nontonal languages (eg, English). Finally, investigations of the effects of linguistic elements (such as the lexical status, syllable structure, number of syllables, and syllable position) on children’s pitch-variation skills or speech motor control will provide information on how the linguistic elements of Cantonese interact with speech motor planning and programming skills.

This study faces several challenges. First, data collection started in January 2022 but was disrupted owing to the fifth wave of the COVID-19 pandemic in Hong Kong. Restrictions on face-to-face interactions during the fifth wave forced the cessation of data collection for several months. Although the fifth wave is now over, the parents of participants are still concerned about mask-off activities during data collection, resulting in slow progress in data collection. Second, the study is recruiting either patients with CAS with an existing CAS diagnosis or individuals suspected of having CAS by a qualified speech-language pathologist. However, a recent study has shown that about half of the Hong Kong speech-language pathologist respondents to a questionnaire (36/77, 47%) had never worked with children with CAS or suspected CAS. Furthermore, a majority of the respondents (64/77, 83%) rated their understanding of Cantonese CAS as “a little” or “fair” [43]. This may be because of a possible low prevalence of CAS in Hong Kong or limited understanding of CAS among local clinicians. Both factors could have limited participant recruitment in this study. In an effort to solve these problems, the research team has provided continuing education opportunities for local speech-language pathologists to enhance their understanding of CAS among Cantonese speakers. In addition, the research team may extend participant recruitment to Macau, another special administrative region of China, because, as in Hong Kong, the people of Macau also use Cantonese as their primary language for oral communication. Third, coexisting developmental issues in the participants may limit the results of this study. Owing to the challenge of recruiting participants with CAS, the research team will not control for coexisting developmental conditions in the participants, such as intellectual disability and ASDs, which may affect the speech production skills of the participants. The research team is aware of this limitation and will balance the sample size and coexisting developmental conditions of the participants.

### Dissemination Plan

This project will benefit the field of speech-language pathology locally and internationally. Locally, the results of this study will be shared with speech-language pathologists through continuing education seminars and conferences. Owing to the limited understanding of CAS in Cantonese speakers, the professional training of local speech-language pathologists currently does not adequately address this severe pediatric SSD. Postqualification continuing education is frequently requested. A study has shown that an understanding of CAS in Cantonese speakers is lacking. Even experienced speech-language pathologists are not confident in using criteria for making a differential diagnosis of CAS in Cantonese-speaking children [44]. The challenge of making such an accurate diagnosis will be ameliorated through the dissemination of these results. If this study finds that the 2 existing diagnostic tools (ie, MPT and SRT) and the potential tool (ie, TST) are effective in differentiating Cantonese-speaking children with CAS from those without CAS, local clinical practices will benefit directly from the study findings and the related assessment package. speech-language pathologists will be more confident in diagnosing children with CAS and providing appropriate treatment, subsequently improving the quality of life of children with CAS and their families. In addition, the local professional training of speech-language pathologists will be enhanced with more data from the local population. The benefit will further extend to society as a medium-term impact, when the appropriate amount of speech-language therapy time is allocated according to valid CAS diagnoses.

Internationally, the results of this study will be shared with other academics through publications in international peer-reviewed journals with open-access and via international conferences. We anticipate that there will be an increased understanding of pitch-variation skills in Cantonese-speaking children with CAS. We also hope to provide a potential diagnostic tool for CAS. This will serve as the basis for further investigations of pitch-variation skills in children who speak other tonal languages, such as Mandarin, Vietnamese, and Thai, and may lead to the development of the TST as a diagnostic tool for CAS in children learning these languages. The results of this study may also provide insight into pitch-variation skills in children with CAS, regardless of their language background. Given that the same underlying deficits in speech motor planning and programming skills manifest differently among different languages [14], this project will have theoretical implications that will impact future international investigations. Ballard et al [21] stated that “exploring additional speaking contexts would be valuable in fully understanding how control of f0 develops over time in children.” If the results show that degraded pitch-variation skills are one of the deficits in children with CAS, the TST can be applied to speakers of English (or other nontonal languages) in an out-of-stress context. This application may shed light on the role of pitch control in out-of-stress contexts and confirm the existence of deficits in pitch control in speakers with CAS across languages.

### Future Investigations

The results of this study will also provide a basis for further investigations of pitch-variation skills in other disordered populations, such as ASDs, hearing impairment, developmental language disorders, acquired apraxia of speech, and aphasia. In the long term, theoretical knowledge about pitch-variation skills in disordered populations will be acquired and applied by frontline health care professionals. In addition to the MPT and SRT, the TST will become a vital component of the assessment process for children with communication disorders.

## Appendix

Multimedia Appendix 1 Peer-review reports from the General Research Fund and Early Career Scheme Research Grants Council of the Hong Kong Special Administration Region Government.

## Abbreviations

ASD: autism spectrum disorder
CAS: childhood apraxia of speech
F0: fundamental frequency
LI: language impairment
MFD: maximum fricative duration
MPT: maximum performance tasks
MRRtri: maximum repetition rate for trisyllabic stimuli
S&LI: speech and language impairment
SRT: Syllable Repetition Task
SSD: speech sound disorder
TD: typical development
TST: tone sequencing task

## Data Availability

The data sets generated and analyzed during this study are not publicly available but can be obtained from the corresponding author on reasonable request.
