# Supplementary material for: The Use of Segmental and Suprasegmental Sequencing Skills to Differentiate Children With and Without Childhood Apraxia of Speech: Protocol for a Comparative Accuracy Study
Source: JMIR Res Protoc. 2022 Oct 4;11(10):e40465. doi: 10.2196/40465 (PMC9579924; doi:10.2196/40465)
Supplement: Multimedia Appendix 1 [file resprot_v11i10e40465_app1.pdf]

Research Grants Council 研究資助局

General Research Fund and Early Career Scheme - Principal Investigator [mwong55] (Change Scheme / Role)

Logout

VIEW COMMENTS FROM PANEL

Project Number : 15605821
Project Title : From segmental sequencing to pitch-variation skills: New insights for diagnosing childhood apraxia of speech
PI Name : Dr WONG Min Ney

It is well designed and nicely written project with convincing preliminary data from a PI having strong track records in the field.

Return

General Research Fund and Early Career Scheme - Principal Investigator SCREEN ID: PM_COMMENT

Research Grants Council 研究資助局

General Research Fund and Early Career Scheme - Principal Investigator [mwong55] (Change Scheme / Role)

Logout

VIEW COMMENTS FROM EXTERNAL REVIEWER

Project Number : 15605821
Project Title : From segmental sequencing to pitch-variation skills: New insights for diagnosing childhood apraxia of speech
PI Name : Dr WONG, Min Ney
Co-I Name : Mr WONG, Eddy Chun-ho
Prof Velleman, Shelley

Section A : Detailed Comments

1. Please comment on the objective(s) of the proposal, and whether the research agenda adequately addresses the objective(s)?

Excellent	Very Good	Good	Fair	Poor
◉	○	○	○	○

Comments:

The overall aim is apparent from the project title: to research a method to assess segmental sequencing and pitch-variation skills in Cantonese-speaking children for diagnosing childhood apraxia of speech. The statement of 3 more specific objectives expands on this overall aim appropriately, The research agenda shows clearly how to address the objectives, with details of experiment design and expected results.

2. Please comment on the Research Design and Methodology.

Excellent	Very Good	Good	Fair	Poor
○	◉	○	○	○

Comments:

The research design and methodology is well presented, starting from an overview of related work which informs the proposed research. The work-plan is based on a well-designed experimental setup, with clearly defined target participants, procedure, and data analysis methods.
My only criticism of the research design and methodology is that it is perhaps "too safe", not going far beyond known versions of MPT, SRT and TST tasks asking the children to say and/or repeat specified phones and words. It would be more adventurous and challenging to investigate whether the child's natural spontaneous speech could be analysed to derive metrics equivalent to MPT, SRT and TST scores.

3. Please comment on the feasibility of the proposed research.

Excellent	Very Good	Good	Fair	Poor
◉	○	○	○	○

Comments:

The research design is clearly laid out and specified in detail, so is clearly feasible if the research team follow the plan.

4. What do your consider to be the most original or innovative aspect of the proposed research? What advances would the research result bring about to the related field if the proposed research is successful?

Comments:

The most original aspect of the proposed research is the extension of apraxia tests to tonal languages, not only Cantonese but potentially other tonal languages such as Thai and Vietnamese. This will enable diagnosis of more children with apraxia of speech; and may have wider application in phonetics research.

5. Please comment on the reasonableness of the proposed budget and manpower planning and project duration.

Comments:

Te proposed budget, manpower and project duration are reasonable, given the time and specialised expertise required to carry out the experiments.

6. Overall Comments

Overall Comment :

A clear research design, with a practical target outcome: to develop and evaluate a new test for apraxia suited to Hong Kong.

Strength:

Clear research design and methodology, straightforward to implement.

Weaknesses:

The types of tests do not go far beyond established tests; it would be more adventurous and challenging to investigate children's natural spontaneous speech directly as a source of data for testing.

Suggested improvements:

ADD as an additional phase of research, the investigation of natural speech as an alternative data-source for possible tests.

Section B : Summary of Assessment

The project :

**Scientific/scholarly merit**	**Excellent**	**Very Good**	**Good**	**Fair**	**Poor**
	◉	○	○	○	○
**Duration Proposed**	**Too Long**	**Appropriate**	**Too Short**		
	○	◉	○		
**Impact of Research**	**High**	**Moderate**	**Low**	**None**	
	○	◉	○	○	

The principal investigator :

**Ability to undertake the proposal**	**Excellent**	**Very Good**	**Good**	**Fair**	**Poor**
	◉	○	○	○	○
**Track record in field**	**Excellent**	**Very Good**	**Good**	**Fair**	**Poor**
	◉	○	○	○	○

Return

Research Grants Council 研究資助局

General Research Fund and Early Career Scheme - Principal Investigator [mwong55] (Change Scheme / Role)

Logout

VIEW COMMENTS FROM EXTERNAL REVIEWER

Project Number : 15605821
Project Title : From segmental sequencing to pitch-variation skills: New insights for diagnosing childhood apraxia of speech
PI Name : Dr WONG, Min Ney
Co-I Name : Mr WONG, Eddy Chun-ho
Prof Velleman, Shelley

Section A : Detailed Comments

1. Please comment on the objective(s) of the proposal, and whether the research agenda adequately addresses the objective(s)?

Excellent	Very Good	Good	Fair	Poor
◉	○	○	○	○

Comments:

Childhood apraxia of speech (CAS) is a very important issue and one that clearly is under researched. This proposal addresses this clear gap in clinical research in the context of tone languages and to base this on addressing the gap for Cantonese seems a logical next step. The outlined research agenda seems to pick up the agenda outlined well in a series of logical steps.

2. Please comment on the Research Design and Methodology.

Excellent	Very Good	Good	Fair	Poor
○	◉	○	○	○

Comments:

The three stated aims seem to produce a logical process for this research:
To show that Maximum Performance Tasks and Syllable Repetition Task can contribute to the diagnosis of childhood apraxia of speech in Cantonese-speaking children,
To document differences in pitch-variation skills in Cantonese-speaking children with versus without childhood apraxia of speech, and
To prove that Tone Sequencing Tasks are effective for diagnosing childhood apraxia of speech in Cantonese-speaking children.

The first hypothesis that 'MPT and SRT will differentiate Cantonese-speaking children with CAS from those without CAS' seems a good starting point given the prevalence of these tasks within normal diagnoses. Pitch variation is clearly a vital part of speech, particularly in the context of a tone language such as Cantonese where it is a vital component so the potential for using Tone Sequencing Tasks seems an appropriate methodology herein.

My only (minor) reservation is what happens if these lines of exploration fail to find a strong contributory link for the relevant diagnoses; what's the back-up plan? What new line of investigation might be looked at?

3. Please comment on the feasibility of the proposed research.

Excellent	Very Good	Good	Fair	Poor
○	◉	○	○	○

Comments:

The fact that the research is based on existing well-established techniques provides confidence in the plan - one does not have to imagine what might happen if the tests do not work because they are established. This is positive in terms of feasibility of the work. The investigators bring strong backgrounds to the work and the therapists are familiar with the tasks; all of these facts lead me to the conclusion that this work is highly feasible and should start producing results rapidly.

The subject recruitment seems to be a potential issue (it will be very challenging to recruit 172 participants within 2 years) for which the total number is reduced to 120. It is not clear how confident the team is in recruiting the 120 but looking at related numbers in other work as stated as '20 to 30 children with CAS for investigations of assessment and diagnostic accuracy' the number look potentially useful.

4. What do your consider to be the most original or innovative aspect of the proposed research? What advances would the research result bring about to the related field if the proposed research is successful?

Comments:

The linking of existing techniques to a new potential analysis - the fact that the tests are already in place and carried out regularly. Any childhood speech disorder is going to hold back all sorts of aspects of communication and development for that child since speech is basic to human existence and getting on in life. This project is addressing this for a specific group of children for whom it is know that the condition has major effects of their communication skills in practice and the further development of those skills.

5. Please comment on the reasonableness of the proposed budget and manpower planning and project duration.

Comments:

The budget seems to me to be well thought through and appropriate for the programme of work proposed.

6. Overall Comments

Overall Comment : This is well thought out as a research proposal which is set in a context where a big difference could be made for young lives.

Strength: The research addresses a community where communication skills could be transformed.

Weaknesses: Subject numbers are potentially concerning but this is often an issue in such research.

Suggested improvements: none

Section B : Summary of Assessment

The project :

**Scientific/scholarly merit**	**Excellent**	**Very Good**	**Good**	**Fair**	**Poor**
	○	◉	○	○	○
**Duration Proposed**	**Too Long**	**Appropriate**	**Too Short**		
	○	◉	○		
**Impact of Research**	**High**	**Moderate**	**Low**	**None**	
	◉	○	○	○	

The principal investigator :

**Ability to undertake the proposal**	**Excellent**	**Very Good**	**Good**	**Fair**	**Poor**
	◉	○	○	○	○
**Track record in field**	**Excellent**	**Very Good**	**Good**	**Fair**	**Poor**
	◉	○	○	○	○

Return

Research Grants Council 研究資助局

General Research Fund and Early Career Scheme - Principal Investigator [mwong55] (Change Scheme / Role)

Logout

VIEW COMMENTS FROM EXTERNAL REVIEWER

Project Number : 15605821
Project Title : From segmental sequencing to pitch-variation skills: New insights for diagnosing childhood apraxia of speech
PI Name : Dr WONG, Min Ney
Co-I Name : Mr WONG, Eddy Chun-ho
Prof Velleman, Shelley

Section A : Detailed Comments

1. Please comment on the objective(s) of the proposal, and whether the research agenda adequately addresses the objective(s)?

Excellent	Very Good	Good	Fair	Poor
⦿	○	○	○	○

Comments:

The proposal seeks to define and refine the diagnostic criteria for CAS in Cantonese-speaking children. This goal will serve both our theoretical knowledge of how CAS manifests across languages, and serve the clinical groups treating children with CAS who speak tone languages. The application is solid, and a good method to attain this goal.

2. Please comment on the Research Design and Methodology.

Excellent	Very Good	Good	Fair	Poor
⦿	○	○	○	○

Comments:

The research design is well-aligned with the goals of the proposal. The team has the necessary expertise to accomplish the research goal.

3. Please comment on the feasibility of the proposed research.

Excellent	Very Good	Good	Fair	Poor
○	⦿	○	○	○

Comments:

There are some concerns regarding the power, as the team proposes to collect fewer children than their power analysis suggested. The team explains this by matching their numbers to the proposed timeline; however, the number of children to be recruited remains highly ambitious, and there is a concern as to whether a sufficient number of children will be recruited in the research period.

4. What do your consider to be the most original or innovative aspect of the proposed research? What advances would the research result bring about to the related field if the proposed research is successful?

Comments:

The most original aspect is the team's goal to develop TST criteria specific to tone language speaking children. Successful completion of the proposed research will result in enhanced criteria to diagnose CAS in Cantonese speaking children, which will be of high benefit to the field of speech pathology.

5. Please comment on the reasonableness of the proposed budget and manpower planning and project duration.

Comments:

I have no concerns regarding budget or personnel.

6. Overall Comments

Overall Comment :
The proposed research's goal will be of benefit to both the theoretical and clinical communities within speech pathology. The team is qualified for the proposed work, indeed, they have already conducted pilot studies to justify the proposed research. The data collection and analysis plans are sound and aligned with the goals of the study.

Strength:
The team is highly qualified for this work. The clinical need for this research is high and the proposed research is solid, with a strong potential to meet the clinical need.

Weaknesses:
There are some concerns regarding the sample size. The proposed sample, while highly ambitious, is smaller than suggested by the power analysis. At the same time, the ambitiousness of the sample raises concerns whether a sufficient number of children will be recruited in the proposed time period.

Suggested improvements:

Greater justification of the sample relative to power and indication of sources for recruiting children would alleviate some of the concerns.

Section B : Summary of Assessment

The project :

**Scientific/scholarly merit**	**Excellent**	**Very Good**	**Good**	**Fair**	**Poor**
	(•)	( )	( )	( )	( )
**Duration Proposed**	**Too Long**	**Appropriate**	**Too Short**		
	( )	( )	(•)		
**Impact of Research**	**High**	**Moderate**	**Low**	**None**	
	(•)	( )	( )	( )	

The principal investigator :

**Ability to undertake the proposal**	**Excellent**	**Very Good**	**Good**	**Fair**	**Poor**
	(•)	( )	( )	( )	( )
**Track record in field**	**Excellent**	**Very Good**	**Good**	**Fair**	**Poor**
	( )	(•)	( )	( )	( )

Return

General Research Fund and Early Career Scheme - Principal Investigator SCREEN ID: ER_COMMENT

Research Grants Council 研究資助局

General Research Fund and Early Career Scheme - Principal Investigator [mwong55] (Change Scheme / Role)

Logout

VIEW COMMENTS FROM EXTERNAL REVIEWER

Project Number : 15605821
Project Title : From segmental sequencing to pitch-variation skills: New insights for diagnosing childhood apraxia of speech
PI Name : Dr WONG, Min Ney
Co-I Name : Mr WONG, Eddy Chun-ho
Prof Velleman, Shelley

Section A : Detailed Comments

1. Please comment on the objective(s) of the proposal, and whether the research agenda adequately addresses the objective(s)?

Excellent	Very Good	Good	Fair	Poor
○	◉	○	○	○

Comments:

The objectives are clear. It would be greater if the project provides theoretical contributions explicitly to the existing literature.

2. Please comment on the Research Design and Methodology.

Excellent	Very Good	Good	Fair	Poor
◉	○	○	○	○

Comments:

Well designed. The data collection seems challenging, but the PI might have preexisting networks related to this project.

3. Please comment on the feasibility of the proposed research.

Excellent	Very Good	Good	Fair	Poor
○	◉	○	○	○

Comments:

The design and method seem a little bit ambitious, but it would be great if this study is conducted since the study has control and comparison groups.
Good luck with the project and am looking forward to reading the related publications in the discipline.

4. What do your consider to be the most original or innovative aspect of the proposed research? What advances would the research result bring about to the related field if the proposed research is successful?

Comments:

The bilingual environment in Hong Kong needs this kind of study and the outcomes of the study will bring great benefits to society.

5. Please comment on the reasonableness of the proposed budget and manpower planning and project duration.

Comments:

The project will last for two years, but the last 6 months are for only conference presentations, preparation of manuscripts, and delivery of seminars and these do not need a sole period without other project activities. Others are reasonable.

6. Overall Comments

Overall Comment :
The bilingual environment in Hong Kong needs this kind of study and the outcomes of the study will bring great benefits to society.

Strength:
Well designed. The data collection seems challenging, but the PI might have preexisting networks related to this project.

Weaknesses:

The project will last for two years, but the last 6 months are for only conference presentations, preparation of manuscripts, and delivery of seminars and these do not need a sole period without other project activities.

Suggested improvements:
It would be greater if the project provides theoretical contributions explicitly to the existing literature.

Section B : Summary of Assessment

The project :

**Scientific/scholarly merit**	**Excellent**	**Very Good**	**Good**	**Fair**	**Poor**
	◉	○	○	○	○
**Duration Proposed**	**Too Long**	**Appropriate**	**Too Short**		
	◉	○	○		
**Impact of Research**	**High**	**Moderate**	**Low**	**None**	
	◉	○	○	○	

The principal investigator :

**Ability to undertake the proposal**	**Excellent**	**Very Good**	**Good**	**Fair**	**Poor**
	○	◉	○	○	○
**Track record in field**	**Excellent**	**Very Good**	**Good**	**Fair**	**Poor**
	○	◉	○	○	○

Return
